# Alpha-Amylase Activity in Feline Saliva: An Analytical Validation of an Automated Assay for Its Measurement and a Pilot Study on Its Changes Following Acute Stress and Due to Urinary Tract Pathologies

**DOI:** 10.3390/ani15142074

**Published:** 2025-07-14

**Authors:** Esmeralda Cañadas-Vidal, Alberto Muñoz-Prieto, Juan D. García-Martínez, Jose J. Ceron, Luis Pardo-Marín, Asta Tvarijonaviciute

**Affiliations:** 1 Interdisciplinary Laboratory of Clinical Analysis Interlab-UMU, Regional Campus of International Excellence ‘Campus Mare Nostrum’, University of Murcia, Calle Campus Universitario 16, Espinardo, 30100 Murcia, Spain; esmeralda.canadas@um.es (E.C.-V.); alberto.munoz@um.es (A.M.-P.); jjceron@um.es (J.J.C.); lpm1@um.es (L.P.-M.); 2 Veterinary Hospital, University of Murcia, Calle Campus Universitario 16, Espinardo, 30100 Murcia, Spain; 3 Department of Animal Medicine and Surgery, Faculty of Veterinary Medicine, University of Murcia, Calle Campus Universitario 16, Espinardo, 30100 Murcia, Spain; juandi@um.es

**Keywords:** cat, salivary alpha-amylase, feline, saliva, sympathetic activation, validation

## Abstract

Salivary alpha-amylase (sAA) increases in response to stressful stimuli in a number of animal species, and it is considered a biomarker of sympathetic nervous system activation. However, no studies have been performed in which sAA has been measured in cats. The aim of this study was to perform an analytical and clinical validation of a commercially available automated assay for the determination of sAA in feline saliva. For the analytical validation, the precision, accuracy, and lower limit of quantification (LLOQ) were determined. To evaluate its response to acute stress, sAA was evaluated in feline saliva before and after stressful stimuli, consisting of a blood extraction. In addition, the sAA activity was compared between cats suffering from urinary tract pathologies and healthy controls. Analytical validation studies confirmed the method as being precise, accurate, and sufficiently sensitive for the sAA determination in cats. When the response to stress was evaluated, a statistically significant increase was detected in sAA in comparison with its activity before the blood extraction. In addition, cats with urinary tract diseases presented higher sAA activity than controls. The results of the present study indicate that sAA can be measured in feline saliva. This study could contribute to a wider use of the measurements of sAA in the saliva of cats and serve as a basis for future investigations aiming to assess acute stress in this species in a non-invasive manner.

## Simple Summary

Salivary alpha-amylase (sAA) increases in response to stressful stimuli in different animal species, indicating the activation of the sympathetic nervous system. However, sAA has never been measured in cats. Therefore, the aim of this study was to perform an analytical and clinical validation of a commercially available automated assay to evaluate its suitability for the determination of sAA in feline saliva. The obtained results indicate that sAA can be accurately measured in feline saliva using an automated commercially available method. Furthermore, when the response to stress was evaluated, a significant increase was detected in the sAA activity in comparison with its levels before the blood extraction. In addition, cats with urinary tract pathologies presented higher sAA activity than healthy controls. Therefore, the findings of this study indicate that sAA can be measured in feline saliva and that it can have a potential use as a biomarker of stress.

## 1. Introduction

Saliva is an oral fluid rich in proteins and analytes that can act as biomarkers to assess both local and systemic diseases [1]. Saliva collection is easy to perform and is non-invasive and painless; therefore, in humans it was suggested as an alternative to blood collection for the measurement of selected analytes, especially in patients highly susceptible to pain, such as children and elderlies [2,3,4,5]. In animals, saliva has been used as a source of biomarkers in stress studies due to its non-invasive nature and the reduced stress of collection compared to blood sampling. Besides its utility to assess and study stress, there are a number of reports indicating saliva being a valuable sample that provides information in different pathologies in different animal species, including dogs, pigs, or horses [6,7]. Nevertheless, and despite the fact that blood collection in cats is a highly stressful situation, to the best of the authors’ knowledge, no studies have been reported validating and measuring salivary analytes in this species.

Salivary alpha-amylase (sAA) is considered a biomarker of the sympathetic nervous system (SNS), and its activity increases in response to stressful stimuli. Therefore, its behavior has been studied by different authors in stress-related and welfare studies in humans [8,9] and different animal species [10,11,12,13,14,15]. For instance, in horses, sAA values were reported to be significantly increased after an acute stress stimulus and in acute abdominal disease [10,11]. Stressful stimuli were also related with an increased sAA activity in pigs, after temporary restraint using a nasal snare or loop, and sheep, after being immobilized and confronted with a sheepdog [12,13]. In dogs, the response of sAA to ejaculation was studied, since it is known to produce sympathetic activation, showing a 2-fold increase in sAA activity after ejaculation [14]. Similarly, a mean 2-fold higher sAA activity was observed in diseased dogs in comparison with healthy controls [15]. Nevertheless, no studies were performed in the saliva of cats, nor do analytical validation studies exist for the measurement of sAA in this species.

The hypothesis of this study was that alpha-amylase can be measured in the saliva of cats, and that this enzyme can show increases in its activity in stressful situations. Therefore, the main aim of this study was to perform an analytical validation of an assay for the measurement of alpha-amylase in the saliva of cats and evaluate the possible changes in this analyte in an acute stressful situation, such as blood sampling. In addition, sAA was evaluated in the presence of urinary tract diseases that have been described to produce stress in cats [16]. In the blood sampling trial and in cats with urinary tract diseases, an estimation of the stress was performed by the Cat Stress Score [17], and the correlation between the results of this score and the sAA activity was studied. The results of the present study provide knowledge on the possibility of the measurement of sAA in the saliva of cats and the potential use of this enzyme as a biomarker to evaluate stress in this species in a non-invasive manner.

## 2. Materials and Methods

### 2.1. Analysis

Salivary alpha-amylase activity was measured using a commercially available automated assay (a-Amylase, OSR6182, Beckman Coulter Inc., Fullerton, CA, USA). This is a spectrophotometric enzymatic assay specifically designed to evaluate alpha-amylase activity. It makes use of a synthetic substrate known as 4,6-ethylidene-(G7)-p-nitrophenol-(G1)-alpha-D-maltoheptaoside (abbreviated as G7PNP). This compound serves as a chromogenic substrate that, when hydrolyzed by alpha-amylase, undergoes an initial cleavage, resulting in the formation of an intermediate oligosaccharide.

This intermediate is subsequently acted upon by the enzyme α-glucosidase, which catalyzes the release of p-nitrophenol, a yellow-colored compound. The appearance of p-nitrophenol is a critical step in the assay, as its concentration directly reflects the extent of alpha-amylase activity in the sample. The more active the alpha-amylase, the greater the amount of intermediate generated and, consequently, the more p-nitrophenol released upon reaction with α-glucosidase.

The p-nitrophenol generated can be monitored and quantified due to its specific absorbance at a wavelength of 405 nm. Therefore, by the measurement of the increase in absorbance at this wavelength over time, the rate of p-nitrophenol formation can be determined. The rate of p-nitrophenol generation is directly proportional to the enzymatic activity of alpha-amylase in the tested sample, and, therefore, the assay provides a reliable and sensitive means of assessing enzyme function under the experimental conditions of the assay. The assay was adapted to an automated analyzer (Olympus 600, Olympus Diagnostica GmbH, Ennis, Ireland), according to the manufacturer’s indications regarding reagent and specimen volumes.

### 2.2. Analytical Validation

The following parameters were assessed to carry out the analytical validation of the method:

- Precision: Two feline saliva samples, one with low and one with high sAA activity, were analyzed. Intra-assay precision was determined by performing five consecutive measurements of each sample within a single analytical run. To evaluate inter-assay precision, each sample was measured once daily over a period of five days. To eliminate any impact from repeated freeze–thaw cycles, samples were stored in individual aliquots, using a fresh aliquot for each measurement. Results were reported as the coefficient of variation (CV), calculated by dividing the standard deviation by the mean of replicates and multiplying by 100%.
- Accuracy: Since no reference method exists for determining sAA activity in cats, accuracy was assessed through dilution linearity. Two samples with distinct sAA activities were serially diluted to concentrations of 75%, 50%, 25%, and 12.5% using deionized water. Measured values were compared to expected concentrations by linear regression analysis.
- Limit of Detection (LD): Defined as the smallest analyte concentration distinguishable from zero. This was calculated by analyzing the zero standard (deionized water) in 10 replicates and determining the mean plus three times the standard deviation.
- Lower Limit of Quantification (LLOQ): Established as the lowest sAA activity that could be reliably quantified above the detection limit, maintaining a CV below 15%. A saliva sample was serially diluted in deionized water, and each dilution was tested in triplicate within the same run. The CV for each dilution was calculated as described above.

### 2.3. Changes in Stress and Disease

The studied animal population included client-owned and animal shelter cats brought to the Veterinary Teaching Hospital of the University of Murcia for periodic revision (healthy animals) or due to the presence of some pathology (diseased animals). In all cases, information about age, gender, breed, body weight, vaccinations, FeLV/FIV testing, and the Cat Stress Score was collected, and a complete physical examination was performed. Cats were not included in the study if they were severely stressed and did not allow themselves to be handled.

BCS was determined using a 9-point scale, where 1 corresponds to extremely underweight and 9 to extreme obesity [18].

Two studies were performed:

- Response to acute stress. To evaluate the dynamics of sAA in feline saliva in response to stress, saliva samples were collected from 21 healthy control animals before and immediately after the blood collection procedure. The included animals were adults (1–22 years), 12 females and 9 males with body weight (BW) between 2.8 and 6.6 kg and body condition score (BCS) of 4–7/9. All cats were healthy (except for overweight in 7 cases) on physical examination and blood analysis. The blood samples were collected for reasons other than the study, e.g., routine check-ups. Blood samples were obtained by the puncture of the jugular vein in all cats.
- Urinary tract diseases. To evaluate the possible changes in sAA in saliva of diseased cats, salivary samples were obtained from healthy and diseased animals with chronic kidney disease (CKD) and feline lower urinary tract disease (FLUTD). Healthy cats (Control Group) were the same as those included in study 1 (response to stress); the sample used was obtained before blood collection.

CKD group was composed of 12 adult animals (4 females and 8 males), with an age range from 2 to 15 years, BW 2.3–6.8 kg, and BCS 2–7/9, that were diagnosed with CKD. The diagnosis was performed based on clinical signs, blood and urine analysis, and abdominal ultrasound results [19].

FLUTD was composed of 14 adult animals (14 males), with an age range from 1 to 10 years, BW 3.0–8.0 kg, and BCS 3–9/9, that were diagnosed with FLUTD based on clinical signs, blood and urine analysis, and abdominal ultrasound results [19].

None of the cats showed external clinical signs of dehydration at the time of sampling, and all samples were obtained between 8 and 10.00 a.m. to minimize the influence of circadian rhythms.

The stress of all animals was evaluated based on a 7-level Cat Stress Score (CSS), were 1 represents fully relaxed animals and 7 represents terrorized animals [17,18,19,20].

### 2.4. Saliva Sample Collection

In all cases, saliva was obtained prior to blood to avoid any possible influence of stress associated with blood collection on the saliva results. Saliva samples were obtained by using the previously reported method for dogs [21]. In brief, a piece of a sponge was introduced in the mouth of an animal to be chewed until it was completely moist, which took around one minute. Afterwards, the sponge was placed into collection devices (Salivette, Sarstedt, Aktiengesellschaft & Co, Nümbrecht, Germany). After centrifugation (3000× *g* for 10 min at 4 °C), the saliva samples were transferred to polypropylene vials (microcentrifuge tube 1.5 mL; Daslab, Barcelona, Spain) and stored at −80 °C until analysis. In all cases, the analyses were performed within two weeks after sample collection.

### 2.5. Statistical Analysis

Statistical analysis was performed using routine descriptive statistical procedures and software (Graph-Pad Version 8 Software, Inc., San Diego, CA, USA; SPSS 15.0, SPSS Inc., Chicago, IL, USA). The sAA data were evaluated for normality of distribution by using the Shapiro–Wilk normality test statistics. As data were not normally distributed, the Wilcoxon test was used to compare data obtained before and after blood extraction. Correlations between variables were estimated using the Spearman correlation coefficient. Differences between different groups of animals were evaluated using the Kruskal–Wallis test followed by Dunn’s multiple comparisons test. All results of *p* < 0.05 were considered significant.

## 3. Results

### 3.1. Analytical Validation

**Figure 1:**
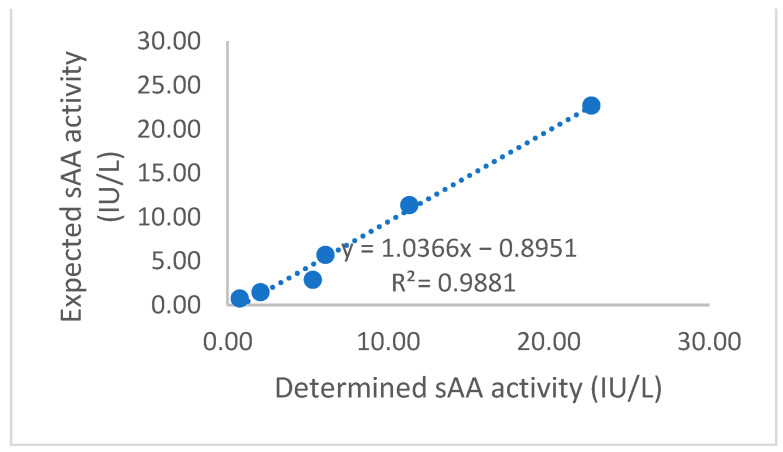
Representative graph of linearity under dilution study of salivary alpha-amylase (sAA) in feline saliva.

**Figure 2:**
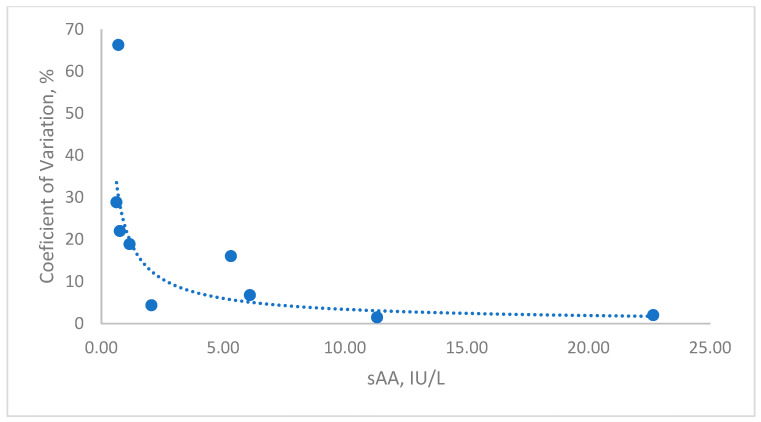
A representative graph of the limit of quantification for the detection of the salivary alpha-amylase (sAA) activity in feline saliva.

Intra- and inter-assay CVs for sAA were <8% for specimens with a low and high sAA activity (Table 1). The linearity under the dilution study yielded a coefficient of correlation close to one in the range from 0.7 to 100 UI/L (Figure 1). The LD was 1.56 U/L, and the LLOQ was 1.65 U/L (Figure 2).

### 3.2. Response to Acute Stress

**Figure 3:**
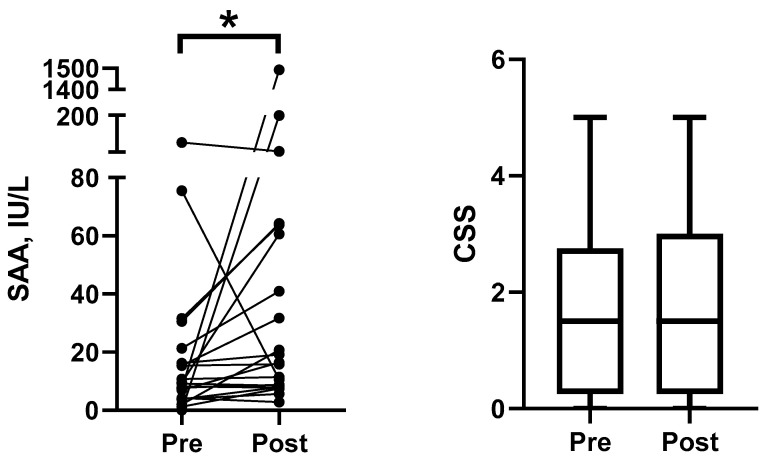
The salivary alpha-amylase (sAA) activity and Cat Stress Score (CSS) from healthy cats (*n* = 21) before and after the blood collection procedure. The boxes depict the median (horizontal line) and interquartile range (top and bottom of box), and the whiskers show the range. *, *p* < 0.05.

When sAA was evaluated before and after the blood extraction procedure, a statistically significant increase was detected in sAA (median (25–75% percentile), 16.4 (8.3–62.2) IU/L) in comparison with its levels before the procedure (9.2 (4.1–18.8) IU/L) (*p* = 0.012) (Figure 3). In contrast, no statistically significant changes were detected for the CSS between the two sampling points (*p* = 0.703).

### 3.3. Changes in Urinary Tract Diseases

**Figure 4:**
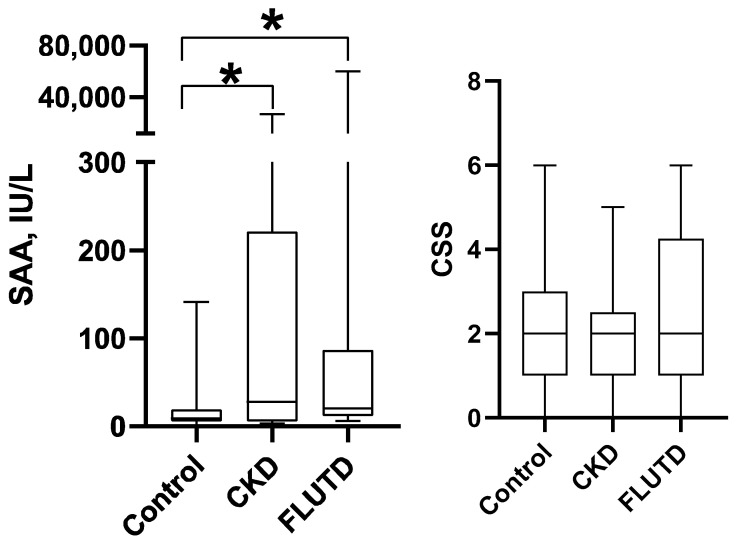
The salivary alpha-amylase (sAA) activity and Cat Stress Score (CSS) in healthy cats (Control Group; *n* = 21) and cats with urine tract diseases (CKD; *n* = 12) and feline lower urinary tract disease (FLUTD; *n* = 14) included in the study. The boxes depict the median (horizontal line) and interquartile range (top and bottom of box); the whiskers show the range. *, *p* < 0.05.

Statistically significant changes were not detected in the CSS between healthy cats and cats with CKD or FLUTD (Figure 4). While, when sAA was assessed, the Kruskal–Wallis test revealed statistically significant differences between different groups of cats (*p* = 0.038). Dunn’s multiple comparison test showed that median (25–75% percentile) sAA levels were statistically significantly higher in cats within the CKD group (27.6 (6.1–221.6) IU/L) and within the FLUTD group (20.3 (11.9–86.6) IU/L) compared to healthy cats (*p* < 0.05 in both cases) (Figure 4).

## 4. Discussion

Saliva is considered a suitable sample for evaluating stress in animals because it is welfare-friendly, non-invasive, and causes minimal stress to the animal [6]. Stress control is particularly important in cats, as stress, regardless of its origin, has been associated with several gastrointestinal disorders, feline interstitial cystitis, and the suppression of the immune system function, which can lead to the development of an infection [22,23]. Moreover, an objective assessment of stress is essential during welfare evaluations and behavioral studies. In addition to salivary biomarkers, other non-invasive samples, such as hair and nails (claws), have also been investigated in cats, demonstrating the feasibility of measuring stress biomarkers like cortisol levels in these samples [16,24,25]. While hair and nails provide valuable information about stress over longer periods, saliva can offer the advantage of reflecting the animal’s acute stress response at the time of sampling.

Salivary alpha-amylase (sAA) is considered as a reliable and non-invasive biomarker of sympathetic nervous system (SNS) activation in humans, and, currently, the measurement of its activity is widely used for these purposes. The sAA reflects the dynamic reactivity of the SNS under various challenging conditions, and numerous studies have demonstrated that sAA levels exhibit a marked increase in response to both psychological and physiological stressors, including mental stress and physical exercise. sAA can be assessed either by measuring its enzymatic activity or its concentration. In the present study, we measured the enzymatic activity, since this approach is easier to perform using an automated assay, and also it has been reported to offer a greater sensitivity that concentration for evaluating stress responses in other animal species such as horses and dogs [11,14].

In the current study, an automated method employed to determine human sAA was analytically validated, for use with feline saliva samples. This method is commercially available and is based on a spectrophotometric kinetic assay. The assay showed adequate precision data, as the CV was below the permitted 15% limit [23]. These results are similar to previously reported data for human saliva (intra-assay CV, <8%; inter-assay CVs <6%) and different animal species (<10% in all cases) [10,11,14]. Furthermore, the accuracy of the method with feline saliva samples was confirmed by the linearity under the dilution study, producing regression coefficients close to one. Finally, the detection limit obtained for the method was sufficiently low to allow the detection of sAA in saliva from all the animals included in the current study. Overall, these results indicate that the method is precise, accurate and sensitive for the sAA determination in feline saliva samples.

In order to assess the sAA response to acute stress, a blood extraction procedure was used, since in cats, the restraint and the venipuncture itself can be frightening and stressful [26,27]. In this line, different studies were performed to find a way to decrease the stress in cats during blood extraction, as well the guidelines were reported to assure the animals’ welfare and quality of life and to avoid the risks of injury to veterinary staff and even owners [28]. In the present study, in the studied population, most of the cats demonstrated increased sAA, and a 1.8-fold increase was observed in the median sAA activity in cats after the blood extraction. In the same manner, the blood extraction resulted in a mean 2-fold increase in sAA activity in pigs, another species highly susceptible to blood collection [12]. The underlying neuroendocrine mechanism for this response is that the fear and/or pain associated with blood sampling activates the sympathetic nervous system, leading to an increased secretion of adrenalin and noradrenaline. These catecholamines can stimulate the production of the alpha-amylase in the salivary glands, which can be measured in saliva. Overall, the blood extraction procedure can be considered a model that induces an adrenergic response in cats, and sAA could be considered as an objective, non-invasive biomarker of this response in this species. These findings are of interest since they could potentially be translated into clinical decision-making or welfare protocols. For example, it may be beneficial to create the most calming conditions possible for blood sampling in cats that exhibit high sAA responses or to use sAA measurements to evaluate and optimize strategies aimed at reducing stress during blood collection [27].

Diseased cats presented higher sAA levels in comparison with the healthy ones, while statistically significant differences between the two groups were not detected when using the CSS. There was also no correlation between sAA and the CSS. Nevertheless, increased sAA levels in diseased animals are in accordance with the previously reported studies, in which increased levels of sAA were detected in human patients with oral diseases, being related to perceived stress [29]. Furthermore, increased levels of another stress marker, cortisol, were recorded in human patients suffering from systemic pathologies, such as diabetes, autoimmune diseases, or neoplasia among others [30]. In the same way, in veterinary medicine, higher sAA values were detected in diseased horses and dogs, suggesting SNS activation [11,15]. Nevertheless, to the best of the authors’ knowledge, this is the first study to assess the stress-related activation of the sympathetic nervous system in diseased cats via a non-invasive biomarker, and now further studies are needed to clarify the physiopathology of the increases in sAA in diseased cats.

When diseased animals were assessed depending on the disease they suffered from, both groups, cats suffering from CKD and FLUTD, presented statistically significant higher sAA levels in comparison with the healthy ones. These data were in accordance with previous findings, which showed feline interstitial cystitis was related to increased stress markers, serum dopamine, urine serotonin, and multimodal environmental modification scores [31]. In the same line, Kim et al. [16] reported that cats with CKD suffered from chronic stress, as higher cortisol levels were detected in the hair of these animals in comparison with the healthy ones. Nevertheless, a statistical significance was not detected between the two groups when using the CSS. Neither was there a correlation between sAA and the CSS. This divergence could be related to the nature of cats to mask signs of stress or pain possibly as a defense mechanism [32]. Additionally, it should be noted that the CSS was developed to evaluate the responses of caged cats in catteries [16] and, therefore, may not be the best option to evaluate the temperament of cats during routine veterinary examinations or during studies on feline welfare and behavior. Overall, the results of the present study suggest that sAA could be used as a stress biomarker in diseased cats.

This study has various limitations. One is that saliva collection was not possible in aggressive or very stressed animals, which could lead to a selection bias and even compromise the applicability of the sAA measurement in veterinary settings or feline welfare research. Secondly, the number of animals included to evaluate the impact of disease presence was relatively low, limiting the statistical power and generalizability of the findings. While changes in sAA were statistically significant, the CSS did not correlate with sAA levels, raising questions about the specificity of sAA as a standalone indicator of stress. Incorporating more detailed behavioral observations or physiological measures would help strengthen the validity of this biomarker. Furthermore, potential confounding factors, such as the salivary flow rate, were not controlled, which could introduce a bias in the data interpretation and conclusions. It is also important to note that in this report we did not correct the sAA activity for the protein concentration or saliva flow rate. This decision was based on previous reports indicating that such corrections did not enhance, and in some cases reduced, the sensitivity of sAA to detect stress under different conditions, and therefore the use of the uncorrected enzymatic activity was recommended [33]. Therefore, this study should be considered a pilot study, and larger studies involving patients with different diseases and different stages of the disease should be conducted in the future to corroborate the findings of this report.

## 5. Conclusions

Salivary alpha-amylase activity can be measured in cats using an automated spectrophotometric assay with an adequate precision, sensitivity, and accuracy. In addition, the sAA activity increases in cats in situations of acute stress or pain, such as after a blood extraction or with urinary tract disease.

## Figures and Tables

**Table 1:** Intra- and inter-assay coefficients of variation (CVs) of repeated determination of alpha-amylase activity (sAA) in feline saliva samples.

Sample	Mean	Intra-CV%	Inter-CV
High	91.0	3.2	5.3
Low	8.1	4.0	7.9

## Data Availability

The data is available upon reasonable request.
